# Repeated Specific Health Guidance and improvement of metabolic syndrome in middle-aged working men: a longitudinal observational study using modified Poisson regression

**DOI:** 10.1265/ehpm.26-00113

**Published:** 2026-09-16

**Authors:** Hiromi Yoshida, Atsuko Taguchi, Tomonori Okamura, Daisuke Sugiyama

**Affiliations:** 1 Graduate School of Health Management, Keio University; 2 Faculty of Nursing and Medical Care, Keio University; 3 Department of Preventive Medicine and Public Health, School of Medicine, Keio University

**Keywords:** Lifestyle intervention, Specific Health Checkup, Specific Health Guidance, Metabolic syndrome, Repeated intervention

## Abstract

**Background:**

Metabolic syndrome (MetS) is a growing global health concern, and lifestyle interventions are important for preventing MetS and its progression to chronic diseases. Although short-term lifestyle interventions (typically within 12 months) have shown some improvements, clinically meaningful improvements in MetS and cardiovascular disease are achieved only in a subset of participants, indicating that many participants derive insufficient benefit. This study aimed to examine the effectiveness of repeated interventions for individuals who do not achieve sufficient weight loss with a short-term lifestyle intervention, using real-world data from Japan’s health screening system, Specific Health Checkup (SHC) and Specific Health Guidance (SHG), by comparing outcomes between individuals who did not achieve sufficient improvement after the initial intervention and received and did not receive health guidance again in the following year.

**Methods:**

This longitudinal observational study used 2018–2020 individual-level data of men aged 40–65 years from a Japanese health insurance database. Modified Poisson regression was used to investigate whether repeated SHG interventions for individuals who did not achieve sufficient improvement despite receiving SHG in fiscal year 2018 increased the probability of achieving ≥3% weight loss between 2019 and 2020.

**Results:**

The repeated and single intervention groups comprised 952 and 1,373 participants, respectively. No significant difference was observed between the groups in terms of ≥3% weight loss between 2019 and 2020 (16.0% vs. 13.5%; risk ratio [RR] = 1.18; 95% confidence interval [CI], 0.97–1.44, adjusted). Analyses stratified by stage of change showed a significant difference in the contemplation stage between the two groups (≥3% weight loss 2019–2020, 19.7% vs. 13.7%; RR = 1.47; 95% CI, 1.04–2.08).

**Conclusions:**

No significant association was observed between repeated interventions and achieving ≥3% weight loss overall. An exploratory subgroup analysis suggested a possible benefit among individuals in the contemplation stage. These findings should be interpreted cautiously and confirmed in future studies.

**Supplementary information:**

The online version contains supplementary material available at https://doi.org/10.1265/ehpm.26-00113.

## Graphical Abstract

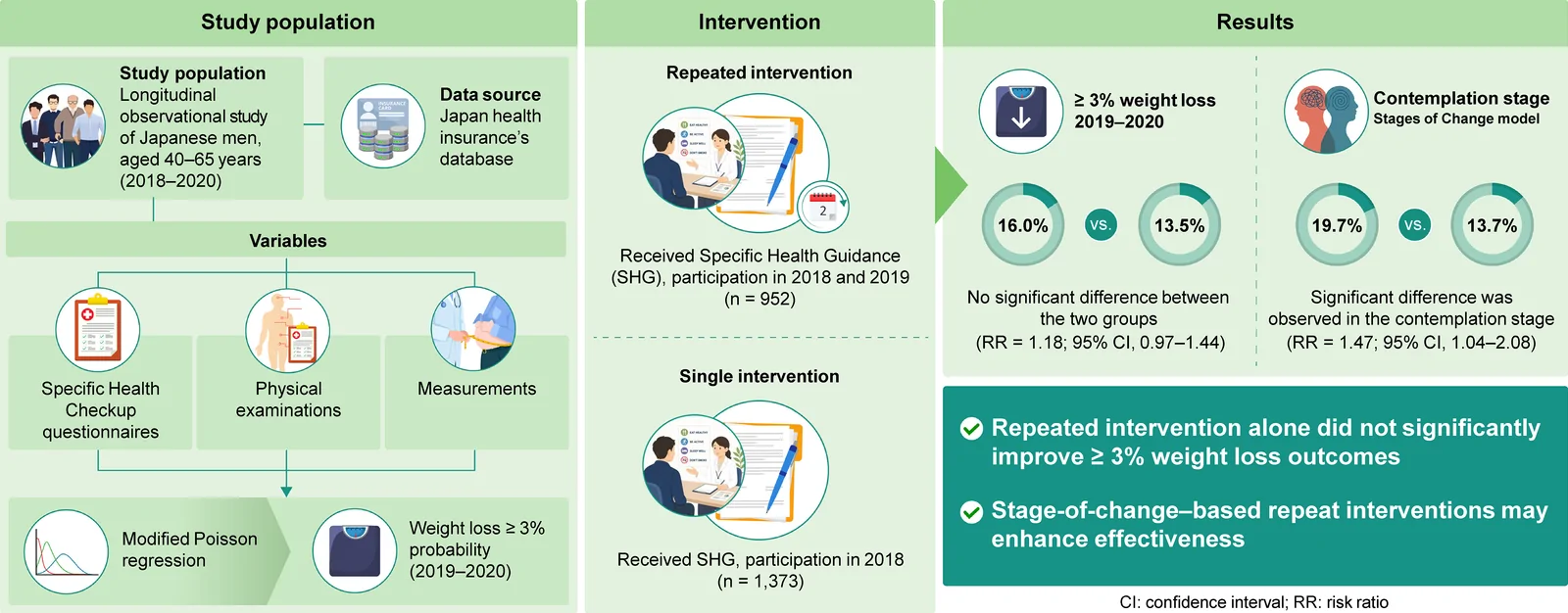

## Introduction

Metabolic syndrome (MetS) is a growing global health concern [1], characterized by abdominal obesity, atherogenic dyslipidemia (elevated triglycerides, low high-density lipoprotein [HDL] cholesterol), raised blood pressure, and insulin resistance (with or without glucose intolerance) [2, 3]. An estimated 1.54 billion adults worldwide are affected by MetS [1], among whom approximately 8.8 million adults (28.2%) in Japan have MetS or are at risk [4]. MetS is a risk factor for chronic and infectious diseases and is associated with elevated healthcare costs [5, 6]. Therefore, proactive management of MetS is important to prevent progression to chronic disease.

Evidence shows that supervised, multifaceted lifestyle interventions improve multiple risk factors and reduce the prevalence of MetS [7]. Consequently, health services should consider implementing such programs, including health guidance and pharmacotherapy, to improve health outcomes and prevent progression to chronic disease [8]. Lifestyle interventions delivered in primary care have also been suggested to be cost-effective [5, 9]. Most lifestyle interventions for MetS are conducted over relatively short intervention periods, generally less than 12 months [8, 10, 11]. Although short-term lifestyle interventions have shown some improvements, clinically meaningful improvements in MetS and cardiovascular disease are achieved only in a subset of participants, indicating that a subgroup derives insufficient benefit [12].

In Japan, health guidance has been implemented to prevent and improve MetS and lifestyle-related diseases prior to the initiation of pharmacotherapy [13]. Specifically, the Japanese health screening system includes Specific Health Checkup (SHC) and Specific Health Guidance (SHG), which allow eligible individuals to be reevaluated annually. Patients diagnosed with MetS or at risk of developing MetS can receive repeated lifestyle improvement guidance. Introduced nationwide in 2008, SHC and SHG target adults aged 40–74 years [13]. SHC focuses on abdominal obesity and cardiometabolic risk factors, and approximately 59.9% (31.23 million) of eligible adults undergo these checkups annually [4]. During middle age, men are more likely to be eligible for SHG than women [14], underscoring the need for targeted support to prevent cardiovascular disease [15]. Individuals diagnosed with MetS or at high risk who opt for SHG receive lifestyle advice and follow-up from health professionals. SHG comprises two components: intensive support for individuals diagnosed with MetS, and motivational support for those at risk of developing MetS. Both services include initial counseling and follow-up evaluations.

During initial counseling, health professionals, including public health nurses, doctors, nutritionists, and trained nurses, explain SHC results, MetS and lifestyle-related diseases, the importance of lifestyle improvement, and the relationship between lifestyle and SHC results to the patients. They also recommend changes such as dietary modification, increased exercise, and smoking cessation, and assist participants in setting actionable goals. These interventions are conducted either in person or online. Intensive support requires at least two follow-up sessions after the initial counseling, delivered in person, online, by phone, or via email. Follow-up evaluations are conducted at least 3 months after reviewing the previous year’s SHC results to assess improvements. Health guidance for individuals aged 65 years or older shifts from preventing lifestyle-related diseases to addressing muscle loss, malnutrition, and frailty [13]. However, whether such guidance is effective for individuals who do not achieve sufficient weight loss through short-term lifestyle interventions and which subgroups or strategies are most effective remains unclear.

Therefore, this study aimed to identify effective and efficient health guidance strategies for individuals who fail to achieve sufficient weight loss through a short-term lifestyle intervention. Using real-world data, we examined the effectiveness of reintervention by comparing the outcomes between individuals who did not improve adequately after an initial intervention and received health guidance again the following year with the outcomes for those who did not receive it, using Japan’s health screening system.

## Methods

### Study design and population

This longitudinal observational study used data from a large-scale database from the Tokyo branch of the Japan Health Insurance Association, containing individual-level data from the SHC collected between April 1, 2018, and March 31, 2020. This study focused on individuals aged 65 years or younger. Given that the priority of health guidance shifts from preventing lifestyle-related diseases to preventing muscle loss, malnutrition, and frailty at age 65, individuals younger than 65 years are a key target population for early intervention [13]. Notably, this study only included male participants because the diagnostic criteria for MetS, patterns of visceral fat distribution, and responsiveness to lifestyle interventions differ substantially between men and women [1, 2, 14]. Furthermore, middle-aged men face higher cardiometabolic risks than women [15], making this group a priority for support.

We evaluated the effects of repeated SHG participation on weight loss among individuals who completed SHG in 2018. Participants were classified into two groups according to the number of times they received SHG: 1) the repeated intervention (RI) group, comprising individuals who received SHG in 2018 and 2019, and 2) the single intervention (SI) group, comprising those who received it only in 2018 (Fig. 1). Changes in SHC results from 2019 to 2020 were then evaluated. Only participants who received intensive support were included in this study. Intensive support represents the most intensive form of standardized lifestyle intervention in SHG and was therefore considered the most appropriate setting for evaluating the effectiveness of continued intervention.

**Fig. 1:**
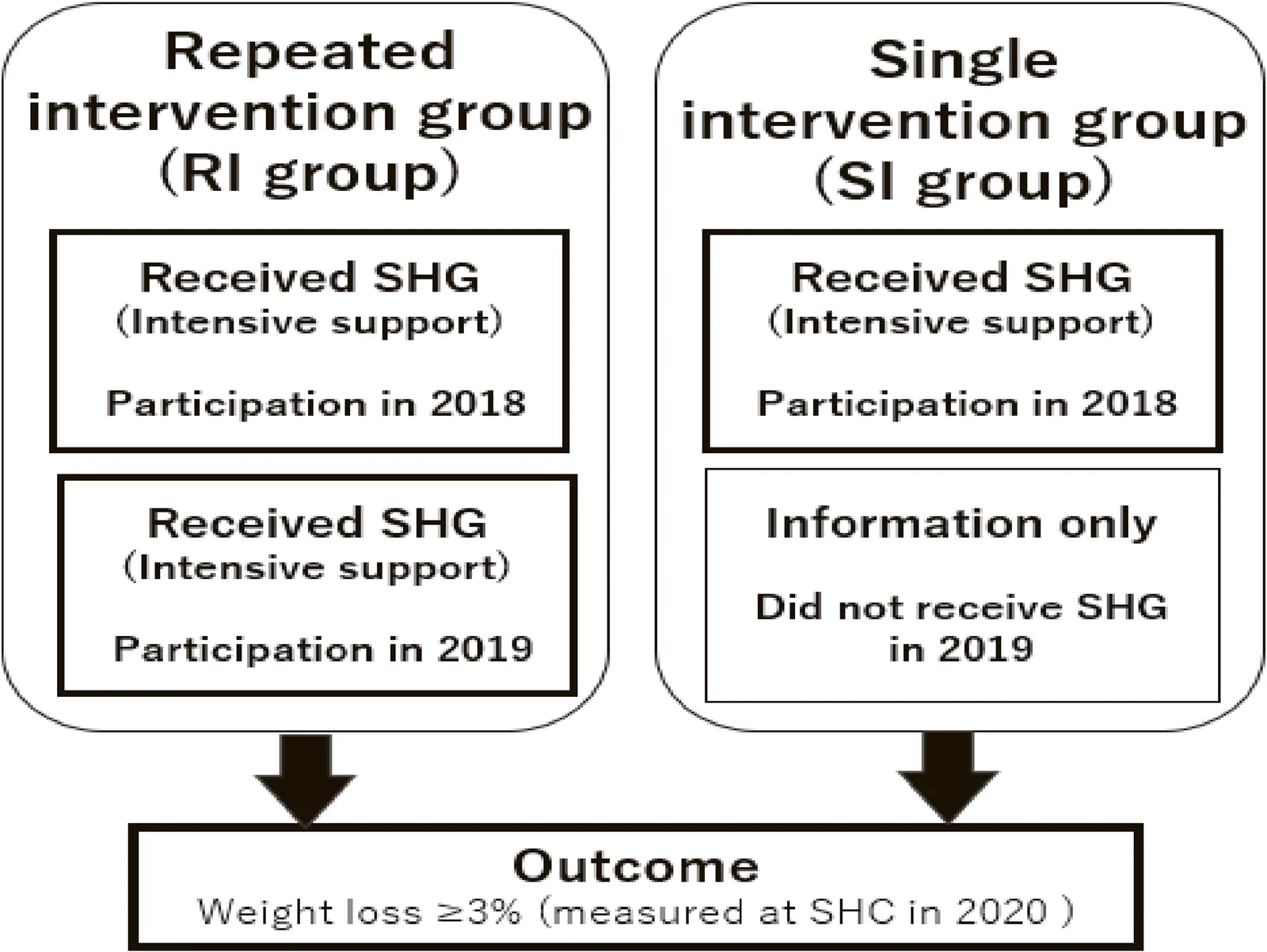
Study design (participation, intervention, and outcome)

The primary outcome was defined as achieving a weight loss of ≥3% between 2019 and 2020, which is generally considered the minimum weight reduction required to improve obesity-related risk factors in Japan [16] and other countries [10, 11, 17]. Accordingly, we adopted ≥3% weight loss as the primary outcome in this study. This study was approved by the Research Ethics Review Committee of the Graduate School of Health Management, Keio University (No. 2024-12).

### Measurements in Specific Health Guidance

Health checkups included questionnaires; physical examinations; and measurements of height and weight, waist circumference, blood pressure (systolic blood pressure [SBP] and diastolic blood pressure [DBP]), serum lipid levels (triglycerides and HDL cholesterol), fasting plasma glucose (FPG), and/or glycated hemoglobin (HbA1c). HbA1c values were expressed according to the National Glycohemoglobin Standardization Program (NGSP). All examinations were conducted under a prescribed ordinance of the Ministry of Health, Labour and Welfare (MHLW) in Japan [13].

Questionnaires captured the history of present and past illnesses, medication history, lifestyle information, and the transtheoretical model (TTM) of behavior change. Smoking status was defined as current smoking or non-smoking. Exercise habits were defined as engaging in at least 30 min of exercise twice or more per week. Physical activity was defined as at least 1 h of walking or equivalent exercise per day. Daily alcohol consumption was defined as habitual drinking. Eating speed was defined as fast if the person perceived their eating speed to be fast. Pre-bedtime dinner was defined as eating dinner before bed at least 3 times per week. Participants who consumed snacks after dinner daily were classified as having post-dinner snacking habits. Those who skipped breakfast three or more times per week were classified as having no habitual breakfast intake. Participants who reported satisfaction with their sleep were classified as having good sleep quality. Stage of change was classified into five stages according to the Stages of Change model: precontemplation (no intention to start within 6 months), contemplation (intention to start within 6 months), preparation (intention to start within 1 month), action (within 6 months of starting), and maintenance (6 months or more after starting) [13].

### Selection criteria for Specific Health Guidance

Individuals who meet the obesity criteria (waist circumference ≥85 cm or body mass index [BMI] ≥25 kg/m^2^) and have two or more additional MetS risk factors are eligible for targeted health guidance (intensive support). The risk factors for MetS are: FPG ≥100 mg/dL or HbA1c ≥5.6%, triglycerides ≥150 mg/dL or HDL cholesterol <40 mg/dL, SBP ≥130 mmHg or DBP ≥85 mmHg, and current smoking. SHG is delivered by healthcare professionals (physicians, public health nurses, and registered dietitians) and involves continuous instruction over at least 3 months on improving lifestyle habits such as exercise, diet, alcohol consumption, smoking, and sleep. After the health checkups, all participants receive brief lifestyle guidance in the form of brochures or other educational materials.

### Statistical methods

Among the baseline participant characteristics (2019), continuous variables are described using mean and standard deviation (SD) or median [25th–75th percentile] depending on the distribution, whereas categorical variables are described using counts (%) to characterize participants at baseline (2019). We compared changes in MetS risk factors from 2019 to 2020 and the proportion of participants who achieved ≥3% weight loss during the same period between the RI and SI groups. Between-group differences were assessed using t-tests or the Wilcoxon rank-sum test for continuous variables and chi-square tests for categorical variables.

Modified Poisson regression was performed to evaluate the association between receiving the standard intervention and achieving ≥3% weight loss at 1 year. This approach was used to avoid overestimation of effect sizes associated with odds ratios [18] because the proportion of participants who achieved ≥3% weight loss was relatively high. Model 1 was an unadjusted regression with ≥3% weight loss at 1 year as the dependent variable. Model 2 was adjusted for age, BMI, and smoking status at baseline as covariates. Model 3 extended Model 2 by adding alcohol consumption, exercise habits, physical activity, eating speed, pre-bedtime dinner, post-dinner snacking, breakfast skipping, sleep quality, and stage of change as covariates. Results are presented as risk ratios (RR) and 95% confidence intervals (CI).

Exploratory subgroup analyses were conducted to examine the following factors previously suggested to influence the effectiveness of health guidance: age [14], previous year improvement status [19, 20], and stage of change (precontemplation, contemplation, preparation, action, and maintenance) [21, 22]. All statistical analyses were performed using R version 4.4.1 (R Foundation for Statistical Computing, Vienna, Austria). Statistical significance was defined as a two-sided p-value < 0.05.

## Results

### Study population

The initial study population included individuals who underwent SHC between 2018 and 2020 (n = 262,796). Among them, individuals who did not undergo SHC in all 3 years (n = 113,800), had implausible values (n = 301), were aged >65 years (n = 7,028), did not participate in SHG (intensive support) in 2018 (n = 74,107), did not complete SHG (intensive support) in 2018 (n = 678), or were ineligible for SHG (intensive support) in 2019 (n = 3,444) were excluded. Among the remaining participants, 1,236 received SHG in 2019, while 1,514 did not. Further exclusions involved individuals who initiated pharmacotherapy (n = 240) or did not complete SHG (intensive support) (n = 185) during the observation period (2019–2020). The final analytic sample comprised 2,325 participants: 952 in the RI group and 1,373 in the SI group (Fig. 2).

**Fig. 2:**
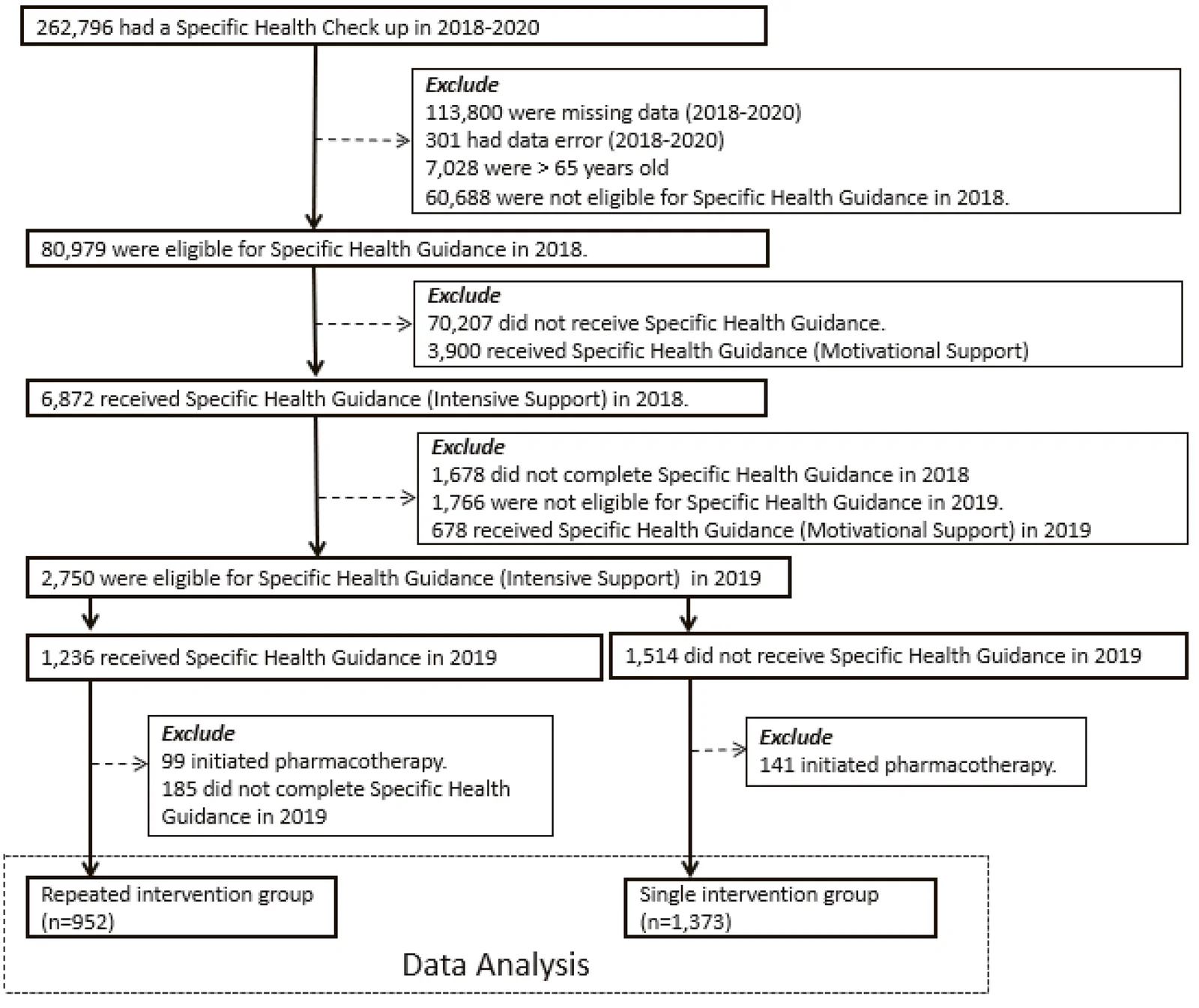
Study population flow diagram

### Baseline characteristics

Table 1 presents the patient baseline (2019) characteristics stratified by the number of SHG sessions. The RI group had a mean age of 51.5 years (SD = 6.40), while the SI group was younger at 50.1 years (SD = 6.39) and included more participants in their 40s (42.1 vs. 51.5%). No significant differences were observed between the two groups in BMI (27.5 vs. 27.4 kg/m^2^), blood pressure (SBP: 130.76 vs. 130.82 mmHg, DBP: 84.64 vs. 84.76 mmHg), triglycerides (187.06 vs. 197.01 mg/dL), HDL cholesterol (50.08 vs. 50.02 mg/dL), FPG (107.89 vs. 106.81 mg/dL), or HbA1c (5.75 vs. 5.73%). However, lifestyle factors differed between the groups: smoking prevalence (59.8% vs. 51.5%), habitual alcohol consumption, and regular physical activity were higher in the SI group than in the RI group. More SI participants achieved ≥3% weight loss during the previous year than RI participants (2018–2019: 17.3% vs. 12.3%).

**Table 1:** Baseline (2019) characteristics of participants

**Variables**	**RI group** **n = 952**	**SI group** **n = 1373**	**p**
Age	51.47 (6.40)	50.08 (6.39)	<0.001
40–49 years	401 (42.1%)	707 (51.5%)	<0.001
50–59 years	419 (44.0%)	540 (39.3%)	
60–65 years	132 (13.9%)	126 (9.2%)	
BMI, kg/m^2^	27.51 (3.51)	27.41 (3.32)	0.492
Weight, kg	80.82 (10.97)	80.88 (10.77)	0.889
Waist circumference, cm	94.65 (7.99)	94.44 (7.59)	0.512
SBP, mmHg	130.76 (14.63)	130.82 (15.27)	0.924
DBP, mmHg	84.64 (10.96)	84.76 (11.43)	0.800
Triglycerides, mg/dL	168 [114–218]	166 [112–228]	0.946
HDL cholesterol, mg/dL	50.08 (11.75)	50.02 (12.24)	0.912
FPG, mg/dL	107.89 (19.61)	106.81 (21.56)	0.224
HbA1c, % (NGSP)	5.75 (0.64)	5.73 (0.66)	0.677
Current smoker	490 (51.5%)	821 (59.8%)	<0.001
Regular exercise	241 (25.3%)	328 (23.9%)	0.461
Physical activity	369 (38.8%)	593 (43.2%)	0.037
Daily alcohol intake	265 (27.8%)	464 (33.8%)	0.003
Fast eating	394 (41.4%)	618 (45.0%)	0.091
Dinner before bedtime	455 (47.8%)	682 (49.7%)	0.396
Daily snacking	134 (14.1%)	140 (10.2%)	0.005
Skipping breakfast	313 (32.9%)	468 (34.1%)	0.574
Sufficient sleep	567 (59.6%)	787 (57.3%)	0.301

Stage of change			0.360
Precontemplation	115 (12.1%)	193 (14.1%)	
Contemplation	284 (29.8%)	371 (27.0%)	
Preparation	208 (21.8%)	291 (21.2%)	
Action	154 (16.2%)	246 (17.9%)	
Maintenance	191 (20.1%)	272 (19.8%)	

Change in BMI (2018–2019)	−0.07 (0.83)	0.04 (0.93)	0.003
Weight loss ≥3% (2018–2019)	117 (12.3%)	237 (17.3%)	0.001

Abbreviations: BMI, body mass index; SBP, systolic blood pressure; DBP, diastolic blood pressure; HDL, high-density lipoprotein; FPG, fasting plasma glucose; HbA1c, glycated hemoglobin; NGSP, National Glycohemoglobin Standardization Program; RI, repeated intervention; SI, single intervention.

### Change in clinical indicators

Table 2 shows the changes from baseline (2019) to the follow-up year (2020) and the proportion of individuals who achieved ≥3% weight loss during this period. Most clinical indicators (BMI, blood pressure, lipid levels, and glucose measures) worsened in both groups; however, the deterioration was smaller in the RI group than in the SI group. During the one-year period, 16.0% of RI participants and 13.5% of SI participants achieved ≥3% weight loss, with no significant difference between them.

**Table 2:** Changes in metabolic syndrome risk factors and ≥3% weight loss from 2019 to 2020

**Variables**	**RI group** **n = 952**			**SI group** **n = 1373**			**p-value for** **difference in** **Δ2020–2019**
	**2019**	**2020**	**Δ2020–** **2019**	**2019**	**2020**	**Δ2020–** **2019**	
BMI, kg/m^2^	27.51 (3.51)	27.55 (3.58)	0.04 (1.11)	27.41 (3.32)	27.57 (3.39)	0.16 (1.12)	0.012
Weight, kg	80.82 (10.97)	80.87 (11.19)	0.05 (3.33)	80.88 (10.77)	81.29 (10.99)	0.41 (3.38)	0.010
Waist circumference, cm	94.65 (7.99)	94.72 (8.38)	0.07 (3.64)	94.44 (7.59)	94.73 (7.88)	0.30 (3.78)	0.148
SBP, mmHg	130.76 (14.63)	132.64 (16.37)	1.87 (12.39)	130.82 (15.27)	133.85 (16.78)	3.02 (12.56)	0.029
DBP, mmHg	84.64 (10.96)	85.73 (11.55)	1.09 (8.67)	84.76 (11.43)	86.76 (12.19)	2.00 (8.71)	0.013
Triglycerides, mg/dL	168 [114–218]	154 [106–224]	−7 [−46–30]	166 [112–228]	158 [112–233]	0 [−40–39]	0.020
HDL cholesterol, mg/dL	50.08 (11.75)	49.99 (11.64)	−0.09 (6.16)	50.02 (12.24)	50.01 (12.16)	−0.01 (6.48)	0.773
FPG, mg/dL	107.89 (19.61)	108.49 (22.84)	0.58 (14.06)	106.81 (21.56)	109.41 (28.25)	2.74 (16.44)	0.001
HbA1c, % (NGSP)	5.75 (0.64)	5.79 (0.86)	0.05 (0.37)	5.73 (0.66)	5.80 (0.86)	0.05 (0.51)	0.812

Weight loss ≥3% (2019–2020), n (%)	152 (16.0%)			186 (13.5%)			0.117

Values are presented as mean (standard deviation [SD]) or median [25th–75th percentile], depending on the distribution. Differences between groups were assessed using t-test for continuous variables, except for triglycerides, which were analyzed using the Wilcoxon rank-sum test. The proportion of participants achieving ≥3% weight loss (2019–2020) was compared using the chi-square test.

Abbreviations: BMI, body mass index; SBP, systolic blood pressure; DBP, diastolic blood pressure; HDL, high-density lipoprotein; FPG, fasting plasma glucose; HbA1c, glycated hemoglobin; NGSP, National Glycohemoglobin Standardization Program; RI, repeated intervention; SI, single intervention; Δ 2019–2020, change from 2019 to 2020 (2020–2019).

### Association between receiving the standard intervention and ≥3% weight loss

Table 3 shows that no significant association was observed between receiving the standard intervention and achieving ≥3% weight loss during 2019–2020 between the two groups (RR ∼ 1.18; 95% CI, 0.96–1.44 across Models 1–3). Subgroup analyses by age and previous year improvement status also showed no differences. However, stratification by stage of change revealed a significant difference in the contemplation stage (RR = 1.43; 95% CI, 1.01–2.03 in Model 1; RR = 1.45; 95% CI, 1.02–2.05 in Model 2; and RR = 1.47; 95% CI, 1.04–2.08 in Model 3; Table 4). In the RI group, 64.4% (n = 183) of those in the contemplation stage in 2019 had also reported being in the contemplation stage in 2018, whereas 17.6% (n = 50) had reported being in the preparation stage. Although an interaction between standard intervention and stage of change was suggested (p = 0.06), no interaction was observed for age (p > 0.7) or previous year improvement status (p > 0.9).

**Table 3:** Modified Poisson regression analysis of factors associated with achieving ≥3% weight loss

**Variables**	**Model 1**		**Model 2**		**Model 3**	
	**RR**	**(95% CI)**	**RR**	**(95% CI)**	**RR**	**(95% CI)**
Group						
Single intervention group	1.00	(Ref.)	1.00	(Ref.)	1.00	(Ref.)
Repeated intervention group	1.18	(0.97–1.44)	1.17	(0.96–1.43)	1.18	(0.97–1.44)
Age, years			1.00	(0.98–1.01)	1.00	(0.98–1.02)
BMI, kg/m^2^			1.04	(1.02–1.07)	1.04	(1.01–1.07)
Non-smoker			1.08	(0.89–1.32)	1.04	(0.85–1.27)
Regular exercise					0.65	(0.50–0.87)
Physical activity					1.12	(0.91–1.38)
Non-daily alcohol intake					1.09	(0.87–1.37)
Non-fast eating					0.80	(0.66–0.98)
No dinner before bedtime					0.97	(0.79–1.19)
No daily snacking					1.06	(0.78–1.45)
Eating breakfast					1.17	(0.94–1.46)
Sufficient sleep					0.97	(0.79–1.19)
Stage of change						
Precontemplation					1.00	(Ref.)
Contemplation					1.39	(0.97–1.99)
Preparation					1.38	(0.95–2.00)
Action					1.24	(0.83–1.85)
Maintenance					1.24	(0.83–1.85)

Model 1: Unadjusted. Model 2: Adjusted for age, BMI, and smoking status. Model 3: Adjusted for Model 2 covariates plus alcohol consumption, regular exercise, physical activity, eating speed, pre-bedtime dinner, daily snacking, breakfast skipping, sufficient sleep, and stage of change.

Abbreviations: BMI, body mass index; RR, risk ratio; CI, confidence interval.

**Table 4:** Stratified analysis of the association between continuing standard intervention and achieving ≥3% weight loss

**Subgroup**	**Weight loss ≥3%** **(2019–2020)** **Events/n (%)**		**Model 1**		**Model 2**		**Model 3**	

	**RI group**	**SI group**	**RR**	**(95% CI)**	**RR**	**(95% CI)**	**RR**	**(95% CI)**
**Age group**								
40–49 years (n = 1108)	73/401 (18.2%)	104/707 (14.7%)	1.24	(0.94–1.63)	1.19	(0.90–1.56)	1.19	(0.90–1.57)
50–59 years (n = 959)	59/419 (14.1%)	64/540 (11.9%)	1.19	(0.85–1.65)	1.17	(0.84–1.62)	1.17	(0.84–1.63)
60–65 years (n = 258)	20/132 (15.2%)	18/126 (14.3%)	1.06	(0.59–1.91)	1.08	(0.60–1.93)	1.23	(0.70–2.14)

**Prior-year weight change (2018–2019)**								
Weight loss <3% (n = 1971)	138/835 (16.5%)	161/1136 (14.2%)	1.17	(0.95–1.44)	1.16	(0.94–1.43)	1.19	(0.96–1.47)
Weight loss ≥3% (n = 354)	14/117 (12.0%)	25/237 (10.5%)	1.13	(0.61–2.10)	1.05	(0.55–2.01)	1.12	(0.59–2.11)

**Stage of change**								
Precontemplation (n = 308)	10/115 (8.7%)	24/193 (12.4%)	0.70	(0.35–1.41)	0.63	(0.33–1.21)	0.59	(0.30–1.17)
Contemplation (n = 655)	56/284 (19.7%)	51/371 (13.7%)	1.43	(1.01–2.03)	1.45	(1.02–2.05)	1.47	(1.04–2.08)
Preparation (n = 499)	36/208 (17.3%)	43/291 (14.8%)	1.17	(0.78–1.76)	1.17	(0.78–1.76)	1.18	(0.79–1.76)
Action (n = 400)	20/154 (13.0%)	37/246 (15.0%)	0.86	(0.52–1.43)	0.93	(0.56–1.54)	0.92	(0.56–1.52)
Maintenance (n = 463)	30/191 (15.7%)	31/272 (11.4%)	1.38	(0.86–2.20)	1.34	(0.83–2.18)	1.37	(0.86–2.19)

The comparison group serves as the reference, and RR (risk ratio) and CI (confidence interval) are calculated using modified Poisson regression. Model 1: Unadjusted. Model 2: Adjusted for age, BMI, and smoking status. Model 3: Adjusted for Model 2 covariates plus alcohol consumption, regular exercise, physical activity, eating speed, pre-bedtime dinner, daily snacking, breakfast skipping, sufficient sleep, and stage of change.

Note: In the analysis stratified by the stage of change, the stage of change variable is excluded from the adjustment covariates.

Abbreviations: RR, risk ratio; CI, confidence interval; RI, repeated intervention; SI, single intervention.

## Discussion

This study examined the effectiveness of reintervention among individuals who did not achieve sufficient weight loss after a short-term lifestyle intervention, using national, real-world data from the SHC and SHG systems in Japan. Our results showed that receiving the standard intervention was not significantly associated with ≥3% weight loss. Nevertheless, this study is the first to examine the effects of reintervention and provides evidence suggesting that reintervention may be effective for specific subgroups. Specifically, exploratory analyses stratified by stage of change indicated that repeated intervention was effective among individuals in the contemplation stage. Based on these findings, we discuss effective approaches for individuals who fail to achieve sufficient improvement through a short-term lifestyle intervention.

Previous studies have reported that short-term lifestyle interventions have shown some improvements in patients with MetS; however, clinically meaningful improvements in MetS and cardiovascular disease are achieved only in a subset of participants, indicating that many participants derive insufficient benefit [12]. Additionally, previous studies have reported that these interventions have limited long-term success, especially at the population level [23], and that achieving clinically significant weight loss is difficult when initial weight loss goals are not met [19, 24, 25]. Our findings are consistent with previous research, suggesting that continuing uniform interventions alone may have limited effectiveness; therefore, intervention strategies should be tailored to individual characteristics.

These results highlight the importance of identifying subgroups for which specialist approaches may be effective. Our findings suggest that classifying participants by stage of change may facilitate the identification of groups most likely to benefit from such approaches. Patient motivation and adherence to recommended lifestyle changes are key determinants of improvement in MetS components [26]. The transtheoretical model (TTM) is widely used to conceptualize health-related behavior change and to help clinicians tailor support according to an individual’s readiness for change [27]. Individuals in pre-action stages, such as the contemplation stage, are more likely to act when provided professional support [28]. Accordingly, our findings suggest that individuals in the contemplation stage were more likely to initiate behavior change and achieve ≥3% weight loss than participants in other stages. Compared with individuals in the precontemplation stage, those in the contemplation stage are more likely to acknowledge that they should change their behavior, although they perceive the pros and cons of behavior change as almost equal, resulting in ambivalence [29]. This creates significant conflict and can cause them to remain in the contemplation stage for an extended period [30]. Previous studies have shown that individuals who receive ongoing professional support demonstrate higher levels of adherence to behaviors recommended by professionals [23].

In contrast, individuals in the precontemplation stage often have limited health awareness and insufficient psychological readiness, and they tend to show little active engagement in behavior modification [22, 26, 31]. Therefore, simply intensifying the intervention at this stage may be insufficient. Rather, enhancing health awareness and supporting consistent engagement in concrete behaviors, such as self-monitoring, may be more closely associated with successful weight loss [26, 32]. These considerations suggest that the evaluation of lifestyle programs should address not only the content of the intervention but also participants’ motivation and adherence, and should incorporate strategies appropriate to their stage of readiness. In our study, the absence of clear effects among participants in the precontemplation stage was consistent with observations reported in previous research. Overall, our findings suggest that professional support may enhance motivation among individuals in the contemplation stage, whereas those in the precontemplation stage are less likely to initiate behavior change than those in more advanced stages. These findings suggest that, rather than applying repeated interventions uniformly to all participants, tailoring intervention strategies according to readiness for change may improve the effectiveness of reintervention.

Previous studies have indicated that participants who do not achieve adequate weight loss through lifestyle interventions are offered meal replacements or pharmacotherapy as adjuncts [19, 33, 34]. In Asia, including Japan, restrictions such as BMI criteria and indications for use are stringent, resulting in a low transition rate to drug therapy [34, 35]. Furthermore, even when drugs are used, herbal medicines are frequently employed, and the use of anti-obesity drugs like glucagon-like peptide-1 receptor agonists remains limited [35]. While medical approaches such as pharmacotherapy have proven effective for individuals who do not respond to lifestyle interventions [36], projections suggest that by 2030, <10% of patients will be able to benefit from pharmacotherapy [37]. Moreover, East Asians, including Japanese populations, tend to accumulate more visceral fat than Western populations and face higher risks of diabetes and cardiovascular disease at lower BMI levels [38–40]. Therefore, identifying effective health guidance strategies and target populations for individuals who do not achieve sufficient weight loss through short-term lifestyle interventions is important.

Given this situation, lifestyle modifications, including weight loss, are crucial for MetS management [8]. Therefore, effective lifestyle guidance tailored to the individual is required. While professional support is effective for improving lifestyle habits [26], prioritization based on factors such as effectiveness and behavioral stage may help optimize the use of limited resources. Consequently, future research is warranted to assess the cost-effectiveness of prioritizing interventions according to readiness for change.

The strengths of this study include its focus on individuals who did not achieve sufficient improvement despite undergoing a standard lifestyle intervention for 1 year, and the examination of characteristics by stage of behavioral change. Notably, these results were obtained from real-world data. To the best of our knowledge, only a few studies have used real-world data to characterize insufficient responses to a single intervention across the stages of behavioral change. Consequently, our results may inform the development of more tailored and effective health guidance strategies for populations that do not respond adequately to a single intervention.

However, this study also had some limitations. First, we did not account for the detailed differences in the intervention content. The MHLW guideline recommends that, if a single intervention proves ineffective, a different intervention should be implemented, such as utilizing information and communication technology [41]. However, in this study, single and repeated interventions were treated as similar interventions occurring prior to the initiation of pharmacotherapy. Therefore, more detailed data on intervention methods and other aspects are needed to examine effective interventions. Second, the study relied on health checkup data from a single Japanese insurance company that covers small- and medium-sized enterprises headquartered in the capital and has subscribers nationwide. Hence, selection bias cannot be ruled out, limiting the generalizability of our findings. Third, self-selection bias may have occurred because participation in SHG was voluntary. Thus, individuals who opted to receive the repeated intervention may have had higher motivation for behavioral change and greater adherence to the intervention, whereas those who did not choose to receive it may have had greater self-efficacy or confidence in managing their health independently. In addition, participants who received repeated interventions may have differed from those who did not in terms of available free time, health literacy, or other unmeasured characteristics. As these factors were not measured and adjusted for in the analysis, their potential influence cannot be excluded. Fourth, reporting bias may be present because smoking status and lifestyle habits were self-reported. Finally, as this was an observational study, the effects of unmeasured confounding factors cannot be ruled out.

## Conclusions

In this study, we examined the effect of repeated interventions in individuals who fail to achieve sufficient weight loss through a short-term lifestyle intervention using Japan’s national real-world data from SHC and SHG. No significant association was observed between receiving repeated interventions and achieving ≥3% weight loss. However, an exploratory subgroup analysis suggested a possible benefit among individuals in the contemplation stage of the Transtheoretical Model. These findings should be interpreted with caution, and further research is needed to determine whether the effectiveness of repeated interventions varies according to the stage of change.

## Abbreviations

MetS: metabolic syndrome
SHC: Specific Health Checkup
SHG: Specific Health Guidance
RR: risk ratio
CI: confidence interval
HDL: high-density lipoprotein
RI: repeated intervention
SI: single intervention
SBP: systolic blood pressure
DBP: diastolic blood pressure
FPG: fasting plasma glucose
HbA1c: glycated hemoglobin
NGSP: National Glycohemoglobin Standardization Program
MHLW: the Ministry of Health, Labour and Welfare
TTM: transtheoretical model
BMI: body mass index
SD: standard deviation

## Supplementary information

**Additional file 1:** The STROBE reporting checklist.

## Declarations

### Availability of data and material

The data that support the findings of this study are not publicly available due to restrictions imposed by the data provider and data use agreements. However, the data are available from the Tokyo Branch of the Japan Health Insurance Association upon reasonable request and with the permission of the data provider.
